# Association Between Dietary Index for Gut Microbiota and Sarcopenic Obesity in Middle‐Aged and Elderly Population: The Mediation Role of Hepatic Steatosis Index

**DOI:** 10.1002/fsn3.71703

**Published:** 2026-03-30

**Authors:** Xiudi Chen, Chenming Liu, Wenwen Fu, Yuxing Liu, Baochun Lu, Haijun Tang

**Affiliations:** ^1^ Department of Hepato‐Biliary‐Pancreatic Surgery Shaoxing People's Hospital Shaoxing China; ^2^ School of Medicine Shaoxing University Shaoxing China; ^3^ The First Affiliated Hospital Shaoxing University Shaoxing China; ^4^ Division of Metabolic and Bariatric Surgery, General Surgery Center Beijing Friendship Hospital, Capital Medical University Beijing China; ^5^ National Clinical Research Center for Digestive Diseases Beijing China; ^6^ Department of Clinical Nutrition The Sixth Affiliated Hospital of Sun Yat‐Sen University Guangzhou China; ^7^ Department of General Surgery, Nanjing BenQ Hospital Affiliated Nanjing Medical University Nanjing China

**Keywords:** dietary index for gut microbiota, hepatic steatosis index, mediation, NHANES, sarcopenic obesity

## Abstract

This study aims to assess the association between the Dietary Index for Gut Microbiota (DI‐GM) and sarcopenic obesity (SO) in middle‐aged and elderly populations, and to evaluate whether the Hepatic Steatosis Index (HSI) acts as the mediation in this relationship. A cross‐sectional analysis was conducted on 3746 participants from the National Health and Nutrition Examination Survey (NHANES) database from 2011 to 2018. Weighted multivariate linear and logistic regression models were used to explore the association between DI‐GM and the prevalence of SO, DI‐GM and HSI, and HSI and the prevalence of SO. Restricted cubic spline (RCS) analysis was used to assess the potential nonlinear relationship between DI‐GM and SO. Subgroup analyses were performed to evaluate the consistency of this relationship across different demographic groups. Additionally, mediation analysis was conducted to explore whether there existed a potential association between DI‐GM, HSI, and SO. Among the 3746 participants included in the study, 369 (9.8%) were diagnosed with SO. After adjusting for all covariates by weighted multivariate logistic regression, each unit increase in DI‐GM was associated with a 15% decrease in the prevalence of SO [Model 3: OR = 0.85, 95% CI (0.76, 0.95), *p* = 0.006]. When DI‐GM was categorized into quartiles, the results remained significant [Model 3: OR = 0.47, 95% CI (0.30, 0.75), *p* = 0.002]. Further analysis indicated that the protective effect of DI‐GM was primarily attributed to the Beneficial gut microbiota score (BGMS). RCS analysis revealed a significant linear relationship between DI‐GM and SO (*p* > 0.05). Subgroup analysis demonstrated the robustness of this association across various subgroups. Mediation analysis showed that 17.8% of the association between DI‐GM and SO was mediated by HSI (*p* < 0.05). DI‐GM is significantly inversely associated with the prevalence of SO in the aging population, and HSI partially mediates this association.

## Graphical abstract

Dietary index for gut microbiota (DI‐GM) is significantly inversely associated with the prevalence of sarcopenic obesity (SO) in the aging population, and hepatic steatosis index (HSI) partially mediates this association. Subgroup analysis demonstrated the robustness of this association across various subgroups.

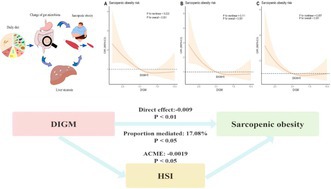

## 1 Introduction

Sarcopenia is an age‐related disease characterized by progressive decline of skeletal muscle mass and function, and it is associated with a series of adverse clinical outcomes, such as frailty, falls, and death (Cruz‐Jentoft and Sayer 2019; Cruz‐Jentoft et al. 2019; Liu et al. 2024). As people age gradually, the total energy consumption of the body also decreases gradually, coupled with a sedentary lifestyle, which not only leads to a reduction in muscle mass but also an increase in body fat. Obesity itself can lead to the loss of muscle mass and function. When obesity and sarcopenia coexist, they often cause more adverse health consequences than either condition alone. Sarcopenic obesity (SO), although not standardized in definition, is generally manifested as the simultaneous existence of the decline of skeletal muscle mass, function and severe obesity (Barazzoni et al. 2018; Donini et al. 2022). Some studies estimated the incidence of SO among middle‐aged and elderly people ranged from 10% to 20%, posing a significant threat to healthcare expenditures worldwide (González Arnáiz et al. 2024). Individuals with SO often exhibited significantly increased incidence of respiratory and cardiovascular system diseases and mortality (Joppa et al. 2016; Farmer et al. 2019; BÅ et al. 2020). Therefore, in order to maintain public health and improve the quality of life for middle‐aged and elderly people, it is of vital importance to effectively prevent and address SO. In recent years, numerous studies have shown that exogenous regulatory diets play a significant role in improving skeletal muscle mass and function (Dennison et al. 2017).

The gut microbiota, as a complex microbial community in the human gastrointestinal tract, plays a crucial role in maintaining the function of the intestinal barrier, regulating nutrients' absorption, participating in metabolism, and regulating the immune system (Fan and Pedersen 2021; Sah et al. 2024). Some basic studies have shown that any alterations in the intestinal community could possibly affect the metabolism of skeletal muscle, thereby influencing muscle mass and function, which confirmed the existence of the so‐called “gut‐muscle axis” (Chen et al. 2022). Diet, as an important exogenous substance for regulating the composition and function of gut microbiota, has always been a research hotspot in recent years (Beam et al. 2021; Ross et al. 2024). In recent years, some scholars have developed the Gut Microbiota Dietary Index (DI‐GM), aiming to evaluate the dietary composition related to healthy gut microbiota (Kase et al. 2024). Recently, studies have shown that a higher DI‐GM score, indicating greater intake of foods beneficial to the gut microbiota, is significantly associated with a reduced incidence of metabolic fatty liver disease and sarcopenia (Wu and Hou 2025; Zheng et al. 2025; Zhang et al. 2025; Li et al. 2025). Several studies have confirmed that the hepatic steatosis index (HSI), as an indicator for evaluating liver metabolic diseases, can be used to predict the occurrence of sarcopenia (Roh et al. 2022). However, SO, as a disease far more complex than sarcopenia, has not yet been evaluated by studies on whether there is a similar association between DI‐GM and it, as well as the mediating role of HSI in this association.

**FIGURE 1:**
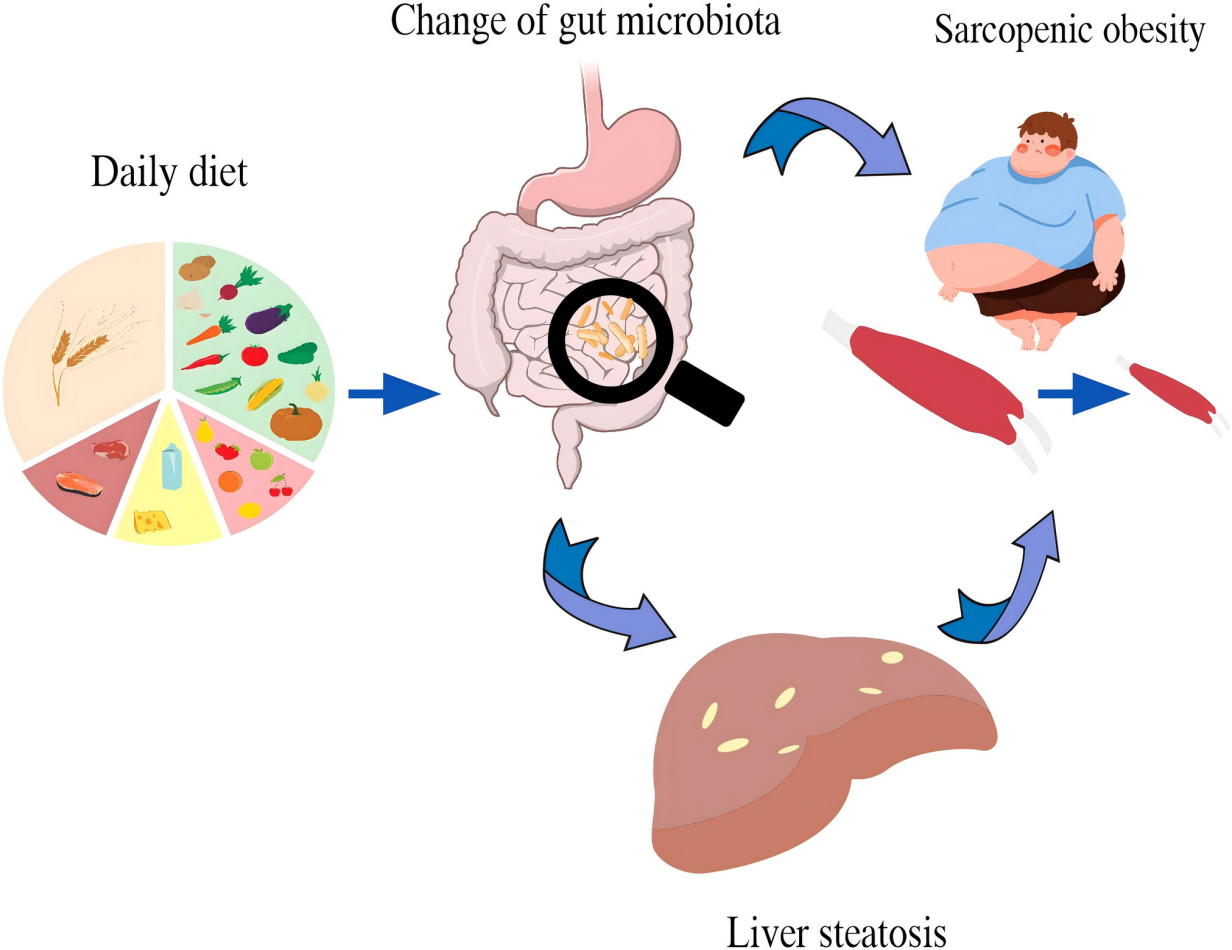
The preliminary schematic diagram of the association between diet, liver steatosis, and sarcopenic obesity.

Therefore, this study aims to evaluate the association between DI‐GM and SO in the middle‐aged and elderly population, and simultaneously explores whether HSI mediates this relationship. Our study hypothesizes that HSI plays a mediating role between the two, which is present in Figure 1 preliminarily.

## 2 Methods

### 2.1 Study Design

This cross‐sectional study utilized data from four consecutive survey cycles (2011–2018) of the National Health and Nutrition Examination Survey (NHANES). NHANES is a nationally representative health and nutrition survey administered by the National Center for Health Statistics (NCHS), designed to assess the health status of the non‐institutionalized U.S. population. All NHANES protocols were approved by the NCHS Research Ethics Review Board, and all participants provided written informed consent before enrollment (Zipf et al. 2013). The study design and all related data analyses were publicly available on the official website (https://www.cdc.gov/nchs/nhanes/).

**FIGURE 2:**
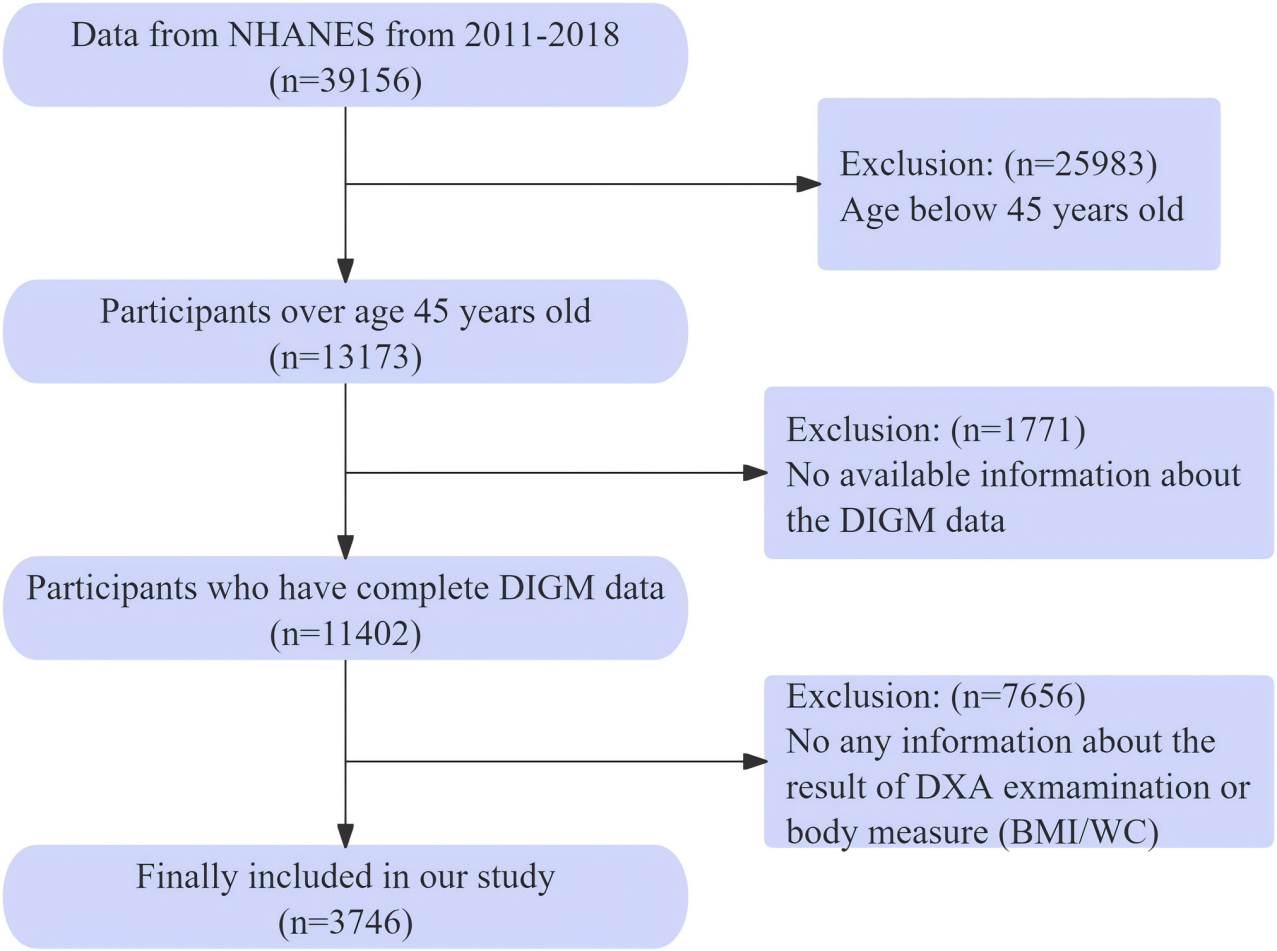
The study flowchart.

Of 39,156 participants, we ultimately identified 3746 participants who were aged 45 years or older with complete DI‐GM and body measurement information including appendicular skeletal muscle index (ASMI), body mass index (BMI), and waist circumference (WC). Participants below 45 years old or with incomplete DI‐GM and body measurement data were excluded. The detailed flowchart was presented in Figure 2.

### 2.2 Evaluation of DI‐GM

DI‐GM, a novel standardized tool for assessing the status of gut microbiota, consists of 14 dietary components: 10 beneficial microbiota components (fermented dairy products, chickpeas, soybeans, whole grains, fibers, cranberries, avocados, broccoli, coffee, and green tea) and four harmful microbiota components (red meat, processed meat, refined grains, and high‐fat diet) (Wu et al. 2025; Liu and Huang 2025). The calculation method of DI‐GM was validated in literature (Kase et al. 2024). Dietary intake was assessed based on the average of two validated 24‐h dietary recalls. The DI‐GM score was calculated based on the gender‐specific median intake of each component, with the exception of high‐fat diet, for which a fixed cutoff of 40% of energy from fat was used. Participants with intake above the gender‐specific median for each beneficial component and below the gender‐specific median for each harmful component were assigned 1 point. Conversely, those with intake below the gender‐specific median for each beneficial component and above the gender‐specific median for each harmful component were assigned 0 points. Individual scores were aggregated into two distinct subscores: Beneficial Gut Microbiota Score (BGMS, range 0–10 points) and Unfavorable Gut Microbiota Score (UGMS, range 0–4 points). The composite score yielded an overall DI‐GM score ranging from 0 to 14 points. The detailed raw data of dietary components used for calculation and respective scores were shown in Tables S1 and S2. For a more comprehensive analysis of the role of DI‐GM, it was transformed into four ranges based on quartile values of the overall population: Q1, Q2, Q3, and Q4.

### 2.3 Evaluation of SO

Our assessment of SO was based on the measurement of sarcopenia and obesity, respectively. For the evaluation of sarcopenia, according to the diagnostic criteria proposed by the Foundation for the National Institutes of Health (FNIH): Appendicular skeletal muscle mass (ASM) was calculated using Dual‐energy X‐ray Absorptiometry (DXA), and ASM was defined as the sum of lean muscle mass (arms and legs), and ASM was divided by body mass index (BMI) to calculate ASMI (kg/m^2^) (Studenski et al. 2014). Sarcopenia was defined as ASMI < 0.789 in men and < 0.512 in women. In accordance with European Society for Clinical Nutrition and Metabolism and the European Association for the Study of Obesity consensus on the assessment of SO, obesity was defined as either BMI ≥ 30 kg/m^2^ or WC exceeding 102 cm for men and 88 cm for women (Donini et al. 2022). Individuals who met both the criteria for sarcopenia and obesity could be considered SO.

### 2.4 Assessment of Several Indicators Reflecting Skeletal Muscle Mass and Obesity

To better reflect the physiological changes associated with the progression of SO (such as metabolic and functional changes), and to identify possible mediators of DI‐GM and SO, we additionally compared Muscle quality index (MQI), Skeletal muscle mass to visceral fat area ratio (SVR), Visceral area index (VAI), Neutrophil to lymphocyte ratio (NLR), C‐reactive protein (CRP), Triglyceride glucose index (TyG), Homeostatic Model assessment‐Insulin Resistance (HOMA‐IR) and Hepatic Steatosis Index (HSI). The HSI was calculated as follows: HSI = 8 × (ALT/AST ratio) + BMI (+2 in women if they are women; +2 in those with diabetes) (Lee et al. 2010). Calculation formulas for other indices were shown in Supporting Files.

### 2.5 Covariates

Covariates included in the analyses were age, sex (male/female), race (Mexican American, non‐Hispanic white, non‐Hispanic black, other Hispanic, and other race), education (less than high school/high school diploma/more than high school), marital status (married/living with partner or widowed/divorced/separated/never married), alcohol consumption status (yes/no), smoking status (never/former/current), and comorbidities including [diabetes, hypertension, heart failure (HF), coronary heart disease (CHD), stroke and cancer]. Alcohol consumption status was obtained from two 24‐h dietary recalls, and participants were defined as drinkers if they reported alcohol consumption during at least one 24‐h dietary recall. Smoking status was assessed by current (< 100 cigarettes), former (never smoked but smoked ≥ 100 cigarettes), or current (≥ 100 cigarettes daily or part of the time). Diabetes was defined according to self‐reported description, use of insulin or anti‐diabetic medications, fasting blood glucose ≥ 126 mg/dL, and glycosylated hemoglobin ≥ 6.5%. Hypertension was defined as mean systolic blood pressure ≥ 140 mmHg and/or diastolic blood pressure ≥ 90 mmHg in four consecutive measurements, a history of anti‐hypertensive treatment, or a self‐reported diagnosis of hypertension. Other comorbidities (HF, CHD, stroke, and cancer) were self‐reported from questionnaires.

### 2.6 Statistical Analyses

To ensure that our analyzed sample accurately represents the general U.S. population, all data were weighted according to NHANES complex survey design. For descriptive statistics, weighted continuous variables were presented as mean ± standard error, and compared between groups using two‐sample *t*‐test. Categorical variables are summarized as counts (*N*) and weighted percentages (%), and differences between groups were assessed using chi‐square tests. Weighted multivariate logistic regression models were employed to examine the association between DI‐GM as both a continuous variable and a categorical variable divided into quartiles (Q1–Q4) and SO (Lumley 2024). For the association between DI‐GM and HSI, the liner regression model was applied. The results were expressed as odds ratios (OR) with 95% confidence intervals (CI). Three regression models were constructed: Model 1: unadjusted for any covariates; Model 2: adjusted for age, sex, education level, race and marital status; Model 3: further adjusted for smoking, alcohol, diabetes, hypertension, CHD, HF, stroke, and cancer. Interaction analyses were conducted to explore potential effect modifications across subgroups subsequently. Restricted cubic spline (RCS) models were used to evaluate nonlinear association in all three models, with further RCS analyses stratified by sex, race, diabetes, hypertension, alcohol, and comorbidities. To assess the mediating role of HSI in the relationship between DI‐GM and SO, regression analyses were first performed, followed by mediation effect estimation using the “mediation” package (Tingley et al. 2013). All analyses were conducted using R software (version 4.4.2). A two‐sided p* value < 0.05 was considered statistically significant.

## 3 Results

### 3.1 General Characteristics of Participants

A total of 3746 participants were included in our study, comprising 1803 males (48.9%) and 1943 females (51.1%), with a mean age of 52.01 ± 4.36. They were divided into two groups: non‐SO group (*N* = 3377) and SO group (*N* = 369). The prevalence of SO in the study population was 9.85%. Table 1 provided a detailed comparison of the basic characteristics between the two groups. We observed that individuals with SO were generally older, predominantly of Non‐Hispanic White and Mexican American ethnicity, less educated, poorer marital status, more comorbidities, and higher DI‐GM and BGMS scores (all differences were statistically significant).

**TABLE 1:** Comparison of characteristics between non‐sarcopenic obesity group and sarcopenic obesity group.

Characteristic	Overall, *N* = 3746	Non‐sarcopenic obesity, *N* = 3377	Sarcopenic obesity, *N* = 369	*p*
Age [Mean (SE)]	52.01 (0.11)	51.87 (0.11)	53.60 (0.33)	< 0.001
Gender, *n* (%)				0.760
Male	1803 (48.9%)	1659 (49.0%)	144 (47.8%)	
Female	1943 (51.1%)	1718 (51.0%)	225 (52.2%)	
Race, *n* (%)				< 0.001
Mexican American	509 (7.2%)	380 (6.0%)	129 (20.5%)	
Other Hispanic	411 (6.1%)	353 (5.7%)	58 (10.3%)	
Non‐Hispanic White	1344 (68.2%)	1230 (69.0%)	114 (59.2%)	
Non‐Hispanic Black	845 (10.7%)	816 (11.3%)	29 (4.1%)	
Other race	637 (7.9%)	598 (8.0%)	39 (5.9%)	
Education_level, *n* (%)				< 0.001
Less than high school	299 (4.2%)	228 (3.4%)	71 (12.6%)	
High school diploma	453 (8.5%)	397 (8.3%)	56 (10.1%)	
More than high school	2993 (87.4%)	2751 (88.3%)	242 (77.3%)	
Marital, *n* (%)				0.014
Married/Living with partner	2426 (69.1%)	2199 (70.0%)	227 (59.6%)	
Widowed/Divorced/Separated/Never married	1319 (30.9%)	1177 (30.0%)	142 (40.4%)	
Alcohol, *n* (%)				0.072
No	2344 (63.0%)	2071 (62.3%)	273 (70.8%)	
Yes	1091 (37.0%)	1026 (37.7%)	65 (29.2%)	
Smoking, *n* (%)				0.609
Never	2074 (54.4%)	1864 (54.6%)	210 (52.8%)	
Former	822 (24.9%)	733 (24.6%)	89 (28.1%)	
Current	850 (20.7%)	780 (20.8%)	70 (19.1%)	
Diabetes, *n* (%)				< 0.001
No	2999 (85.4%)	2755 (86.6%)	244 (72.0%)	
Yes	747 (14.6%)	622 (134%)	125 (28.0%)	
Hypertension, *n* (%)				< 0.001
No	1897 (53.9%)	1750 (55.1%)	147 (40.3%)	
Yes	1849 (6.1%)	1627 (44.9%)	222 (59.7%)	
AIP [Mean (SE)]	−0.11 (0.03)	−0.13 (0.03)	0.22 (0.07)	< 0.001
HF, *n* (%)				0.546
No	3678 (98.6%)	3320 (98.7%)	358 (98.3%)	
Yes	68 (1.4%)	57 (1.3%)	11 (1.7%)	
CHD, *n* (%)				0.033
No	3659 (97.8%)	3306 (98.1%)	353 (95.2%)	
Yes	87 (2.2%)	71 (1.9%)	16 (4.8%)	
Stroke, *n* (%)				0.049
No	3637 (98.0%)	3283 (98.2%)	354 (96.3%)	
Yes	109 (2.0%)	94 (1.8%)	15 (3.7%)	
Cancer, *n* (%)				0.089
No	3475 (90.9%)	3142 (91.2%)	333 (87.6%)	
Yes	271 (9.1%)	235 (8.8%)	36 (12.4%)	
BMI [Mean (SE)]	29.24 (0.18)	28.62 (0.16)	36.16 (0.41)	< 0.001
WC [Mean (SE)]	100.58 (0.42)	99.26 (0.39)	115.11 (1.05)	< 0.001
ASMI [Mean (SE)]	0.78 (0.004)	0.80 (0.004)	0.60 (0.01)	< 0.001
MQI [Mean (SE)]	3.30 (0.03)	3.33 (0.03)	3.03 (0.06)	< 0.001
SVR [Mean (SE)]	0.22 (0.003)	0.23 (0.03)	0.12 (0.003)	< 0.001
VAI [Mean (SE)]	2.07 (0.07)	2.00 (0.07)	2.85 (0.26)	0.004
NLR [Mean (SE)]	2.18 (0.03)	2.17 (0.03)	2.33 (0.06)	0.013
CRP [Mean (SE)]	0.38 (0.03)	0.36 (0.02)	0.71 (0.09)	0.001
TyG [Mean (SE)]	8.73 (0.03)	8.71 (0.03)	9.10 (0.07)	< 0.001
HOMA‐IR [Mean (SE)]	3.87 (0.25)	3.58 (0.22)	7.52 (1.10)	0.001
HSI [Mean (SE)]	38.58 (0.22)	37.9 (0.20)	46.28 (0.51)	< 0.001
DIGM [Mean (SE)]	5.18 (0.05)	5.22 (0.05)	4.77 (0.13)	0.004
BGMS [Mean (SE)]	2.66 (0.04)	2.69 (0.04)	2.33 (0.11)	0.003
UGMS [Mean (SE)]	2.52 (0.03)	2.53 (0.03)	2.44 (0.10)	0.406

Abbreviations: AIP, atherogenic index of plasma; ASMI, appendicular skeletal mass index; BMI, body mass index; CHD, coronary heart disease; CRP, C‐reactive protein; DIGM, dietary index for gut microbiota; HF, heart failure; HSI, hepatic steatosis index; IR, homeostatic model assessment of insulin resistance; MQI, muscle quality index; NLR, neutrophil to lymphocyte ratio; SVR, skeletal muscle mass to visceral fat area ratio; TyG, triglyceride‐glucose index; VAI, visceral area index; WC, waist circumstance.

### 3.2 Association Between DI‐GM and SO

**FIGURE 3:**
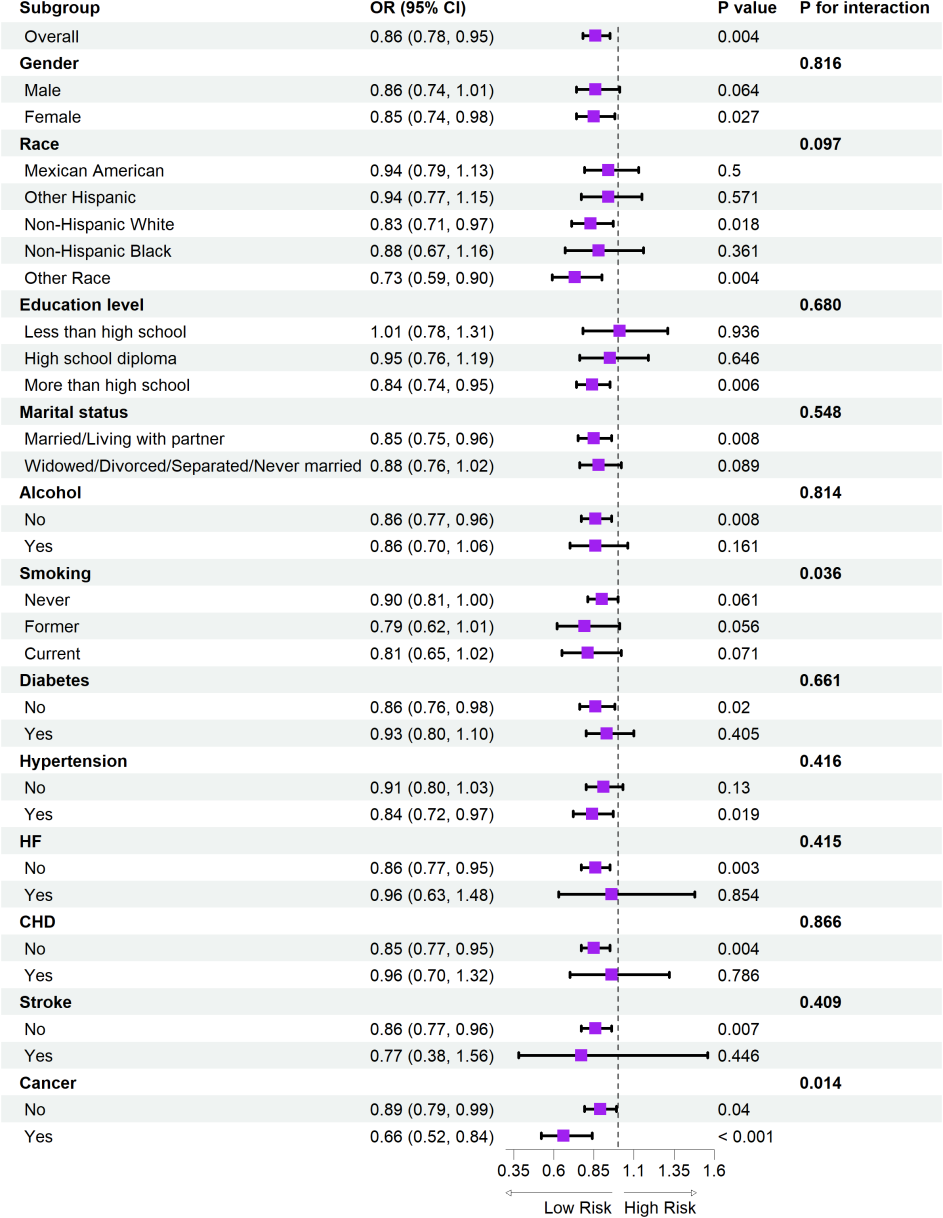
The subgroup analysis of the relationship between DIGM and SO. DIGM, dietary index for gut microbiota; SO, sarcopenic obesity.

Our multivariate regression analysis revealed a significant inverse association between DI‐GM and SO (Table 2), which remained consistent after adjusting for potential confounders [Model 2: OR = 0.84, 95% CI (0.76, 0.94); Model 3: OR = 0.85, 95% CI (0.76, 0.95); all *p*‐values < 0.01]. Furthermore, when DI‐GM was analyzed as a categorical variable by quartiles, this association became more pronounced. Specifically, in Model 3, compared to the lowest quartile (Q1), the prevalence of SO in the highest quartile (Q4) was associated with a 53% reduction [OR = 0.47, 95% CI (0.30, 0.75), *p* = 0.002]. Additionally, we found that the BGMS score was significantly associated with a lower risk of SO [Model 3: OR = 0.85, 95% CI (0.75, 0.95), *p* = 0.007], while UGMS **s**howed no significant association (*p* > 0.05). Subgroup analyses based on covariates demonstrated the stability of this association across all subgroups (nearly all interaction *p*‐values > 0.05). The protective effect of DI‐GM was particularly pronounced in females, Non‐Hispanic Whites, married individuals, **and those** without comorbidities (*p* < 0.05; Figure 3).

**TABLE 2:** Association between DIGM and sarcopenic obesity.

Variable/Model	Model 1		Model 2		Model 3	
	OR (95% CI)	*p*	OR (95% CI)	*p*	OR (95% CI)	*p*
**DIGM‐sarcopenic obesity**						
Continuous	0.86 (0.78, 0.95)	0.004	0.84 (0.76, 0.94)	0.002	0.85 (0.76, 0.95)	0.006
**DIGM (quartile)**						
Q1	1.00 (Reference)		1.00 (Reference)		1.00 (Reference)	
Q2	0.77 (0.51, 1.15)	0.196	0.68 (0.46, 1.02)	0.061	0.75 (0.48, 1.16)	0.189
Q3	0.49 (0.31, 0.76)	0.002	0.43 (0.27, 0.70)	< 0.001	0.41 (0.23, 0.71)	0.002
Q4	0.50 (0.32, 0.77)	0.002	0.45 (0.29, 0.70)	< 0.001	0.47 (0.30, 0.75)	0.002
*p* for trend	0.001		< 0.001		0.002	
**BGMS**	0.85 (0.76, 0.95)	0.003	0.84 (0.75, 0.94)	0.004	0.85 (0.75, 0.95)	0.007
**UGMS**	0.92 (0.77, 1.11)	0.401	0.90 (0.74, 1.09)	0.267	0.90 (0.73, 1.10)	0.289

Abbreviations: DIGM, dietary index for gut microbiota; HSI, hepatic steatosis index; Model 1, unadjusted; Model 2, adjusted for age, gender, race, education level and marital; Model 3, adjusted for age, gender, race, education level, marital, alcohol, smoking, diabetes, hypertension, heart failure, coronary heart disease, stroke and cancer; OR, odds ratio.

### 3.3 Interrelation Between DI‐GM, HSI and SO

Table 3 presented the results of our further regression analysis of the interrelation of DI‐GM, HSI, and SO. We found a significant negative association between DIGM and HSI whether DIGM was used as a continuous or categorical variable [Model 3: β 95% CI: −1.47 (−2.45, −0.48), *p* = 0.004]. The higher the HSI score, the higher the risk of SO [Model 3: OR 95% CI: 1.17 (1.13, 1.21), *p* < 0.001].

**TABLE 3:** Association between DIGM, HSI and sarcopenic obesity in the entire cohort.

Variable/Model	Model 1		Model 2		Model 3	
	*β* (95% CI)	*p*	*β* (95% CI)	*p*	*β* (95% CI)	*p*
**DIGM‐HSI**						
Continuous	−0.45 (−0.65, −0.25)	< 0.001	−0.42 (−0.63, −0.21)	< 0.001	−0.28 (−0.46, −0.07)	0.008
**DIGM (Quartile)**						
Q1	0 (Reference)		0 (Reference)		0 (Reference)	
Q2	−1.01 (−2.09, 0.07)	0.066	−1.01 (−2.08, 0.06)	0.064	−0.91 (−1.91, 0.09)	0.072
Q3	−1.94 (−2.95, −0.92)	< 0.001	−1.94 (−2.98, −0.89)	< 0.001	−1.42 (−2.42, −0.02)	0.007
Q4	−2.17 (−3.18, −1.15)	< 0.001	−2.05 (−3.09, −1.01)	< 0.001	−1.47 (−2.45, −0.48)	0.004
*p* for trend	< 0.001		< 0.001		0.004	
**Variable/Model**	**Model 1**		**Model 2**		**Model 3**	
	**OR (95% CI)**	***p***	**OR (95% CI)**	***p***	**OR (95% CI)**	***p***
**HSI‐sarcopenic obesity**						
Continuous	1.15 (1.13, 1.17)	< 0.001	1.17 (1.14, 1.19)	< 0.001	1.17 (1.13, 1.21)	< 0.001

Abbreviations: DIGM, dietary index for gut microbiota; HSI, hepatic steatosis index; β, regression coefficient; Model 1, unadjusted; Model 2, adjusted for age, gender, race, education level and marital; Model 3, adjusted for age, gender, race, education level, marital, alcohol, smoking, diabetes, hypertension, heart failure, coronary heart disease, stroke and cancer; OR, odds ratio.

### 3.4 RCS Analyses Between DI‐GM and SO

**FIGURE 4:**
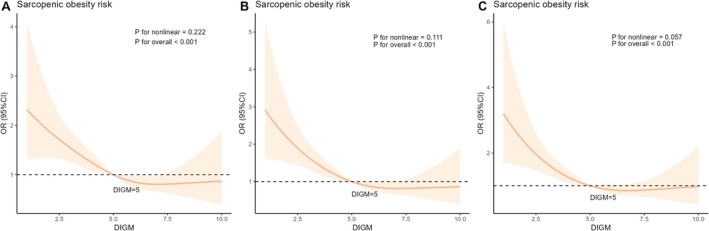
The RCS analyses of the relationship between DIGM and SO. DIGM, dietary index for gut microbiota; RCS, restricted cubic spline; SO, sarcopenic obesity.

**FIGURE 5:**
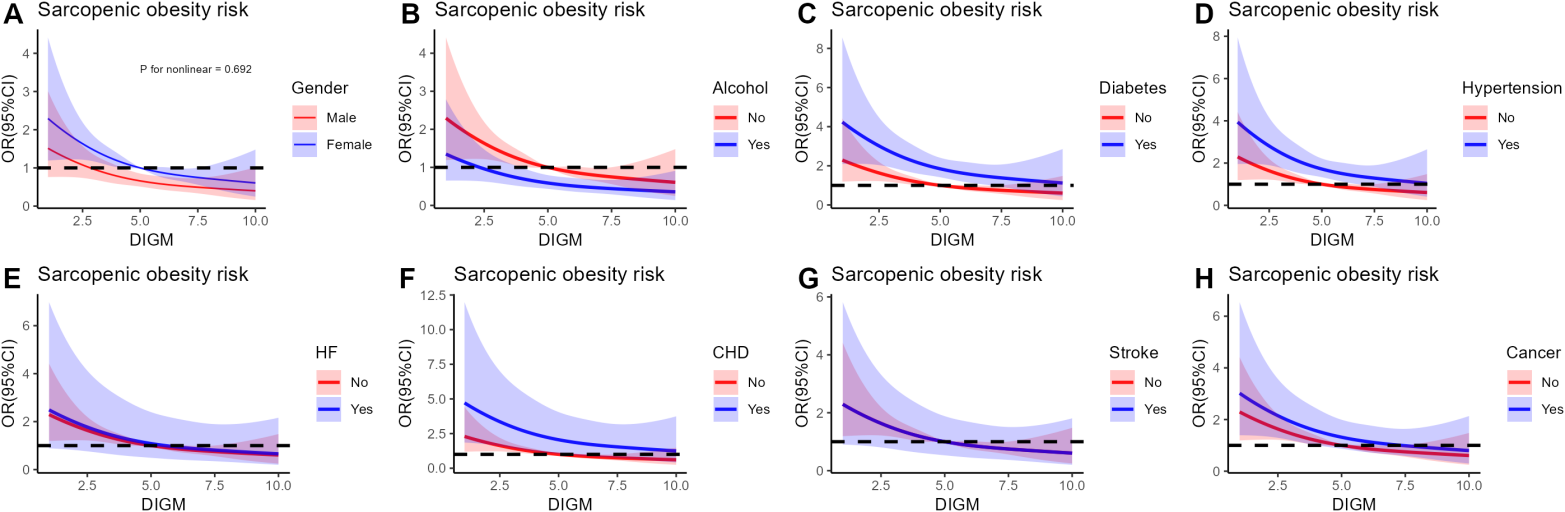
RCS analyses for several subgroups. RCS, restricted cubic spline.

We conducted three RCS analyses between DI‐GM and SO, adjusting for covariates in sequential models. The results showed an inverse linear relationship between DI‐GM and SO in the overall population (all *p‐*values > 0.05 for the three models) (Figure 4). Subsequent subgroup RCS analyses stratified by sex, alcohol, and comorbidities further confirmed the stability of this inverse linear relationship across all subgroups (Figure 5).

### 3.5 Mediation Analysis

**FIGURE 6:**
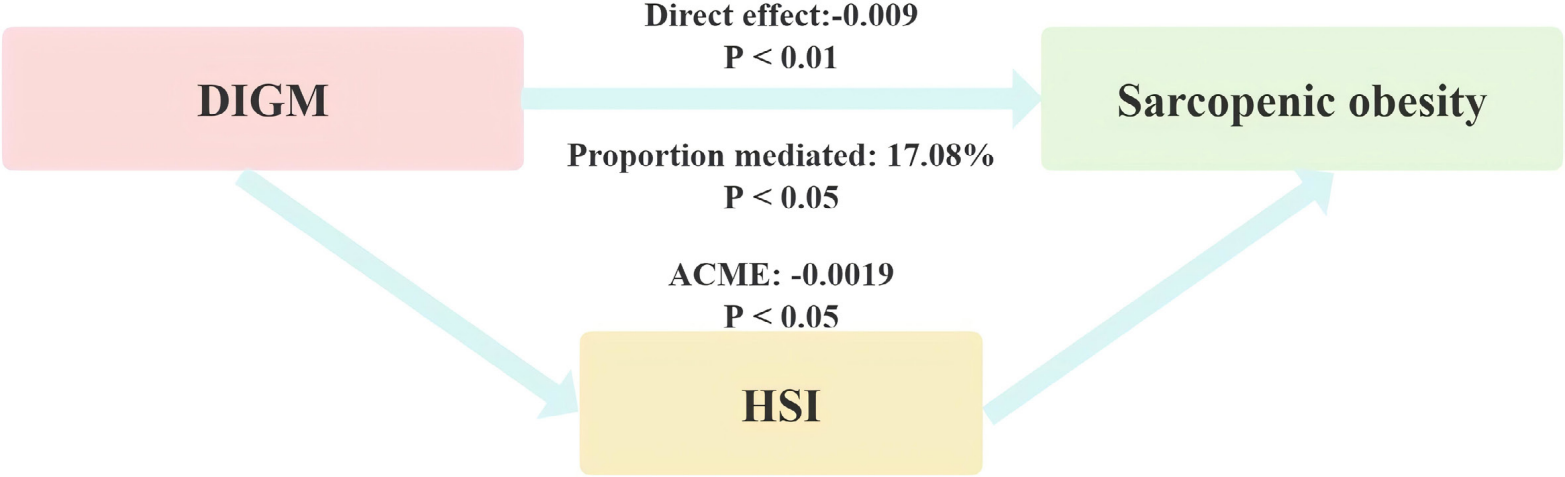
The mediation model between DIGM and SO. DIGM, dietary index for gut microbiota; SO, sarcopeic obesity.

The mediation model and pathway were shown in Figure 6, with DI‐GM as the independent variable, SO as the dependent variable, and HSI as the mediator variable. After adjusting for all covariates, we determined the mediating effect of HSI (direct effect = −0.009, *p* < 0.01; ACME = −0.0019, *p* < 0.05; Proportion mediated: 17.08%). Therefore, HSI can be considered a possible mediator of the association between DI‐GM and SO.

### 3.6 Sensitivity Analysis

In order to demonstrate the robustness of our results, we conducted sensitivity analysis of all statistical results above by including SO population only measured by WC. The results of sensitivity analysis were shown in Supporting Files, which showed the still significant association between DI‐GM and SO (Table S3), the interrelation of DI‐GM, HSI, and SO (Table S4), and HSI as the mediation between DI‐GM and SO (Proportion mediated: 15.74%).

## 4 Discussion

SO, as a relatively novel concept, has garnered increasing attention in recent years. Given that its adverse health impacts—including elevated risks of disability, cardiovascular events, and mortality—far exceed those of either condition alone, reducing the prevalence of SO remains a critical clinical challenge (Joppa et al. 2016; Farmer et al. 2019; BÅ et al. 2020; Batsis et al. 2014). Based on NHANES data from 2011 to 2018, this cross‐sectional study was the first to preliminarily demonstrate that higher DI‐GM scores were significantly associated with a reduced risk of the incidence of SO and to identify HSI as a mediating factor in this relationship. This association remained robust even after adjusting for potential confounders [Model 3: OR = 0.47, 95% CI (0.30, 0.75), *p* = 0.002]. Subgroup analyses further confirmed the consistency of this relationship, with the benefits of DI‐GM being particularly pronounced among females, Non‐Hispanic Whites, married individuals, never‐smokers, and those without comorbidities. Subsequent RCS analyses revealed a linear dose–response relationship between DI‐GM and SO.

Evidence suggests that dietary components influencing gut microbiota are linked to the development of metabolic liver diseases (Valdes et al. 2018). A recent study by Wu et al. demonstrated that higher DI‐GM scores were significantly associated with a reduced risk of metabolic dysfunction‐associated fatty liver disease and metabolic alcohol‐associated liver disease (OR = 0.59, 95% CI: 0.46–0.75; OR = 0.57, 95% CI: 0.46–0.70) (Wu and Hou 2025). The underlying mechanisms by which gut microbiota influence liver steatosis are complex and involve the alterations of intestinal barrier function, effects of fatty acid metabolites, and interactions between epigenetics and the gut microbiome. Among these, the targeting of hepatic adipocytes by metabolites has been relatively well‐studied (Skelly et al. 2019; Tripathi et al. 2018; Ha et al. 2024). Branched‐chain amino acids modulate insulin‐mediated glucose metabolism in hepatocytes by activating enteroendocrine cells via G‐protein‐coupled receptors (GPR41/GPR43), stimulating the secretion of gut hormones (e.g., GLP‐1, PYY), thereby improving insulin sensitivity and suppressing the synthesis of hepatic lipids (Miao et al. 2024). High DI‐GM foods, such as those rich in dietary fiber (e.g., vegetables, whole grains), promote the production of short‐chain fatty acids (e.g., butyrate), which serve as energy substrates for colonocytes, enhance satiety, reduce food intake, and mitigate accumulation of hepatic fat (Kase et al. 2024; Koh et al. 2016). In addition, probiotics (e.g., Firmicutes) in fermented dairy products further promote the production of short‐chain fatty acids, establishing a positive feedback loop in “gut‐liver axis signaling” (Zhou et al. 2019).

Our study also confirmed a strong association between hepatic steatosis and SO, which remained significant after adjusting for confounders [OR = 1.17, 95% CI (1.13, 1.21)]. Similarly, a multi‐center prospective study conducted by Roh et al. in Korea demonstrated that non‐alcoholic fatty liver disease (NAFLD) could predict the development of sarcopenia (Roh et al. 2022). After adjusting for potential confounders, they found that higher HSI levels were associated with an increased risk of low skeletal muscle mass and function [Q4 vs. Q1, OR = 3.46 (2.23–5.35); Q4 vs. Q1, OR = 5.81 (3.67–9.21)]. A 5‐year follow‐up study of individuals aged 20 and older showed that those with NAFLD experienced a significantly greater decline in ASM compared to those without NAFLD (−39.9 g, 95% CI: −53.1 to −26.8, *p* < 0.001) (Sinn et al. 2022). The mechanisms linking hepatic steatosis to SO are not fully elucidated but are thought to involve insulin resistance, chronic inflammation, and vitamin D deficiency, with insulin resistance playing a central role (Polyzos et al. 2023; Kim and Choi 2019). Placental protein A, a physiological inhibitor of insulin receptor tyrosine kinase in skeletal muscle, is upregulated in NAFLD (Mathews et al. 2000). Thus, hepatic steatosis may exacerbate insulin resistance, leading to loss of skeletal muscle mass and function. Conversely, adipose tissue accumulation promotes lipid infiltration and immune cell activation, releasing cytokines (e.g., IL‐6, TNF), adipokines (e.g., leptin), and inducing oxidative stress, collectively worsening insulin resistance and systemic inflammation. This creates a vicious cycle that further accelerates fat accumulation (Polyzos et al. 2017). Two NHANES‐based cross‐sectional studies confirmed that individuals with both NAFLD and sarcopenia had significantly higher all‐cause, cardiovascular, and cancer‐related mortality rates compared to those with NAFLD alone (Golabi et al. 2020; Moon et al. 2021).

In summary, based on the above discussion and our mediation analysis, we concluded that adverse changes in gut microbiota may contribute to hepatic steatosis, which in turn promoted chronic inflammation and insulin resistance, ultimately impairing skeletal muscle mass and function. This cascade leads to reduced the compromised quality of skeletal muscle, loss of homeostasis, and worse clinical outcomes (Ticinesi et al. 2019). Gong et al. (2025) preliminarily demonstrated an inverse association between DI‐GM and sarcopenia, with inflammation acting as a mediator (OR = 0.85, 95% CI: 0.77–0.94). Importantly, our study found that the protective effect of DI‐GM primarily stemmed from BGMS, where higher BGMS scores correlated with a lower incidence of SO, while UGMS showed no significant association. This highlights the importance of consuming “microbiota‐friendly” foods such as fermented dairy, dietary fiber, and whole grains to mitigate SO. We recommend adopting anti‐inflammatory diets and prebiotic‐rich dietary patterns (e.g., Mediterranean diet) to promote beneficial gut microbiota, combined with protein supplementation and resistance training to improve SO. Future prospective studies should focus on elucidating the mechanistic pathways linking gut microbiota, hepatic steatosis, and sarcopenic obesity.

We acknowledge several limitations of our study: First, the nature of cross‐sectional design explicitly stated that strong causality cannot be inferred about the relationship between microbiota‐modulating diets and SO. Large‐scale prospective studies are needed to validate this association. Second, DI‐GM was derived from two 24‐h dietary recalls, which may introduce recall bias. Third, the definition of SO was partially incomplete. For one thing, relying on BMI, which was possibly confounded by low muscle mass, to define obesity in a sarcopenic population was not perfect although the results of subsequent sensitivity analysis were not influenced significantly. For another, due to limited available variables, we were unable to incorporate measures of muscle function (e.g., grip strength) into the diagnosis of SO. Despite these limitations, this is the first study to use a standardized DI‐GM tool to assess the impact of gut microbiota on SO and to identify HSI as a mediating factor in this relationship.

## 5 Conclusions

Our findings preliminarily confirmed a significant inverse association between the DI‐GM and the prevalence of SO, with HSI identified as a mediating factor in this relationship.

## Author Contributions

**Xiudi Chen:** conceptualization, methodology, writing – original draft, software, investigation, formal analysis. **Baochun Lu:** validation, data curation. **Haijun Tang:** writing – review and editing, project administration, supervision, validation. **Wenwen Fu:** conceptualization, methodology, software, investigation. **Chenming Liu:** conceptualization, writing – original draft, methodology, software, formal analysis. **Yuxing Liu:** software, methodology.

## Ethics Statement

The studies involving human participants were reviewed and approved by the National Center for Health Statistics Research Ethics Review Board. All methods were carried out in accordance with relevant guidelines and regulations (Declaration of Helsinki).

## Consent

Informed consent was obtained from all participants involved in the study.

## Conflicts of Interest

The authors declare no conflicts of interest.

## Supporting information


**Table S1:** The detailed raw data of dietary components used for calculation.

**Table S2:** BGMS and UGMS calculated by respective dietary components. BGMS, Beneficial Gut Microbiota Score; UGMS, Unfavorable Gut Microbiota Score.

**Table S3:** Association between DIGM and sarcopenic obesity by sensitivity analysis. DIGM, dietary index for gut microbiota.
**Table S4:** Association between DIGM, HSI and sarcopenic obesity by sensitivity analysis. DIGM, dietary index for gut microbiota; HSI, hepatic steatosis index.

## Data Availability

The data that support the findings of this study are available on request from the corresponding author. The data are not publicly available due to privacy or ethical restrictions.
