# Supplementary material for: Association Between Dietary Index for Gut Microbiota and Sarcopenic Obesity in Middle‐Aged and Elderly Population: The Mediation Role of Hepatic Steatosis Index
Source: Food Sci Nutr. 2026 Mar 30;14(4):e71703. doi: 10.1002/fsn3.71703 (PMC13170585; doi:10.1002/fsn3.71703)
Supplement: Supplementary file 3 — Table S3: Association between DIGM and sarcopenic obesity by sensitivity analysis. DIGM, dietary index for gut microbiota. Table S4: Association between DIGM, HSI and sarcopenic obesity by sensitivity analysis. DIGM, dietary index for gut microbiota; HSI, hepatic steatosis index. [file FSN3-14-e71703-s001.docx]

**Supplementary Table 3. Association between DIGM and sarcopenic obesity by sensitivity analysis**

| **Variable/Model** | **Model 1** | | **Model 2** | | **Model 3** | |
| --- | --- | --- | --- | --- | --- | --- |
|  | **OR (95%CI)** | ***P* value** | **OR (95%CI)** | ***P* value** | **OR (95%CI)** | ***P* value** |
| **DIGM-sarcopenic obesity** | | | | | | |
| Continuous | 0.84 (0.76, 0.93) | 0.001 | 0.82 (0.74, 0.92) | < 0.001 | 0.82 (0.74, 0.93) | 0.002 |
| **DIGM (Quartile)** | | | | | | |
| Q1 | 1.00 (Reference) |  | 1.00 (Reference) |  | 1.00 (Reference) |  |
| Q2 | 0.76 (0.51, 1.14) | 0.180 | 0.68 (0.45, 1.01) | 0.056 | 0.75 (0.48, 1.16) | 0.195 |
| Q3 | 0.48 (0.31, 0.74) | 0.001 | 0.42 (0.26, 0.68) | < 0.001 | 0.40 (0.23, 0.69) | 0.002 |
| Q4 | 0.46 (0.30, 0.72) | < 0.001 | 0.41 (0.26, 0.64) | < 0.001 | 0.43 (0.27, 0.69) | < 0.001 |
| P for trend | < 0.001 | | < 0.001 | | < 0.001 | |
| **BGMS** | 0.83 (0.75, 0.92) | < 0.001 | 0.82 (0.73, 0.92) | < 0.001 | 0.82 (0.73, 0.92) | 0.001 |
| **UGMS** | 0.91 (0.75, 1.10) | 0.334 | 0.88 (0.72, 1.08) | 0.219 | 0.89 (0.72, 1.09) | 0.246 |
| DIGM, Dietary index for gut microbiota; OR, odds ratio; HSI, Hepatic steatosis index; Model 1, unadjusted; Model 2, adjusted for age, gender, race, education level and marital; Model 3, adjusted for age, gender, race, education level, marital, alcohol, smoking, diabetes, hypertension, heart failure, coronary heart disease, stroke and cancer | | | | | | |

**Supplementary Table 4. Association between DIGM, HSI and sarcopenic obesity by sensitivity analysis**

| **Variable/Model** | **Model 1** | | **Model 2** | | **Model 3** | |
| --- | --- | --- | --- | --- | --- | --- |
|  | ***β* (95%CI)** | ***P* value** | ***β* (95%CI)** | ***P* value** | ***β* (95%CI)** | ***P* value** |
| **DIGM-HSI** | | | | | | |
| Continuous | -0.44 (-0.65,-0.25) | < 0.001 | -0.42 (-0.63,-0.21) | < 0.001 | -0.27 (-0.46,-0.07) | 0.008 |
| **DIGM (Quartile)** | | | | | | |
| Q1 | 0 (Reference) |  | 0 (Reference) |  | 0 (Reference) |  |
| Q2 | -1.01 (-2.09,0.07) | 0.066 | -1.01 (-2.08,0.06) | 0.064 | -0.91 (-1.91,0.09) | 0.072 |
| Q3 | -1.93 (-2.95,-0.92) | < 0.001 | -1.93 (-2.98,-0.89) | < 0.001 | -1.42 (-2.42,-0.41) | 0.006 |
| Q4 | -2.17 (-3.18,-1.15) | < 0.001 | -2.05 (-3.09,-1.01) | < 0.001 | -1.47 (-2.45,-0.48) | 0.004 |
| P for trend | < 0.001 | | < 0.001 | | 0.004 | |
| **Variable/Model** | **Model 1** | | **Model 2** | | **Model 3** | |
|  | **OR (95%CI)** | ***P* value** | **OR (95%CI)** | ***P* value** | **OR (95%CI)** | ***P* value** |
| **HSI-sarcopenic obesity** | | | | | | |
| Continuous | 1.15 (1.13,1.17) | < 0.001 | 1.17 (1.14,1.20) | < 0.001 | 1.18 (1.15,1.21) | < 0.001 |
| DIGM, Dietary index for gut microbiota; HSI, Hepatic steatosis index; β, regression coefficient; OR, odds ratio; Model 1, unadjusted; Model 2, adjusted for age, gender, race, education level and marital; Model 3, adjusted for age, gender, race, education level, marital, alcohol, smoking, diabetes, hypertension, heart failure, coronary heart disease, stroke and cancer | | | | | | |

**R code summary:**

1. **Weight application:**

library("survey")

data$WTMEC8YR<-data$WTMEC2YR/4

options(survey.lonely.psu = "adjust")

study_design <- svydesign(data=data,

id=~SDMVPSU,

strata=~SDMVSTRA,

weights=~WTMEC8YR, nest=TRUE)

1. **Regression:**

svyglm( ~ , study_design,family =quasibinomial)

1. **RCS analysis:**

source("rcs_new.R")

library("dplyr")

library("rms")

fit_RCS <- svyglm( ~ rcs(DIGM, ),study_design,family = quasibinomial)

AIC(fit_RCS)

an<-anova(fit_RCS)

an

test_PLOT <- get_rcs(fit_RCS)

1. **mediation analysis:**

library(mediation)

library(lpSolve)

fit.totaleffect<-lm(Group~DIGM+Age + Gender + Race + Education_level + Marital + Alcohol + Smoking + Diabetes + Hypertension + HF + CHD + Stroke + Cancer, data)

summary(fit.totaleffect)

fit.mediator<-lm(HSI~DIGM+Age + Gender + Race + Education_level + Marital + Alcohol + Smoking + Diabetes + Hypertension + HF + CHD + Stroke + Cancer,data)

summary(fit.mediator)

fit.Y<-lm(Group~DIGM+HSI+Age + Gender + Race + Education_level + Marital + Alcohol + Smoking + Diabetes + Hypertension + HF + CHD + Stroke + Cancer,data)

summary(fit.Y)

results <- mediate(fit.mediator, fit.Y, treat='DIGM', mediator='HSI',boot = T)

summary(results)

plot(results)

**Calculation formulas for indices:**

Muscle quality index (MQI):

MQI=handgrip strength /appendicular skeletal mass

Atherogenic index of plasma (AIP):

AIP=ln (TG/HDL-C）

Visceral area index (VAI):

For man: VAI=waist circumference (39.68+1.88×BMI) * TG/1.03*1.31/HDL-C

For women: VAI=waist circumference/(36.58+1.89×BMI) *TG/0.81*1.52/HDL-C

Triglyceride glucose index (TyG):

TyG=ln(triglyceride*glucose/2)

Homeostatic Model Assessment-Insulin Resistance (HOMA-IR):

HOMA-IR=fasting blood glucose×fasting insulin/22.5
